# Development and validation of a nomogram for predicting severity in patients with hemorrhagic fever with renal syndrome: A retrospective study

**DOI:** 10.1515/med-2021-0307

**Published:** 2021-06-25

**Authors:** Zheng Yang, Qinming Hu, Zhipeng Feng, Yi Sun

**Affiliations:** Department of Infectious Disease, Jingzhou Hospital, Yangtze University, Jingzhou, 434020, China; Department of Dermatology, Jingzhou Hosiptal, Yangtze University, No. 60 Jingzhong Road, Jingzhou District, Hubei Province, Jingzhou, 434020, China

**Keywords:** hantavirus, hemorrhagic fever with renal syndrome, severity, nomogram, predictive model

## Abstract

**Background:**

Hemorrhagic fever with renal syndrome (HFRS) is a zoonotic disease caused by hantavirus infection. Patients with severe HFRS may develop multiple organ failure or even death, which makes HFRS a serious public health problem.

**Methods:**

In this retrospective study, we included a total of 155 consecutive patients who were diagnosed with HFRS, of whom 109 patients served as a training cohort and 46 patients as an independent verification cohort. In the training set, the least absolute shrinkage and selection operator (LASSO) regression was used to screen the characteristic variables of the risk model. Multivariate logistic regression analysis was used to construct a nomogram containing the characteristic variables selected in the LASSO regression model.

**Results:**

The area under the receiver operating characteristic curve (AUC) of the nomogram indicated that the model had good discrimination. The calibration curve exhibited that the nomogram was in good agreement between the prediction and the actual observation. Decision curve analysis and clinical impact curve suggested that the predictive nomogram had clinical utility.

**Conclusion:**

In this study, we established a simple and feasible model to predict severity in patients with HFRS, with which HFRS would be better identified and patients can be treated early.

## 1 Introduction

Hemorrhagic fever with renal syndrome (HFRS) is a rodent-borne zoonotic disease caused by hantavirus infection. HFRS can be caused by *Hantaan virus* (*HTNV*), *Dobrava virus* (*DOBV*), *Seoul virus* (*SEOV*), *Amur virus* (*AMV*), *Puumala virus* (*PUUV*), etc. The severity of HFRS patients caused by different viral infections is also different [1]. HFRS is characterized by systemic vascular endothelial dysfunction and increased vascular permeability. The clinical manifestations include fever, hemorrhage, renal insufficiency, thrombocytopenia, and shock [2,3]. HFRS is mainly prevalent in Asia and Europe, while China is the most serious epidemic area in the world. A total of 1,118,124 cases were reported during 2008–2018 in China, which accounts for more than 90% of global HFRS cases [4,5,6]. In China, HFRS is mainly infected by *HTNV* and *SEOV*, and the mortality rate of HFRS caused by these viruses is between 5 and 15%, making it a serious public health concern [7]. Until now, there is no effective antiviral treatment for HFRS, which leads to a high mortality rate in critically ill cases. Early and accurate assessment of the severity and prognosis of HFRS patients is of great significance for guiding clinical treatment and the reasonable allocation of medical resources.

However, currently, there is no simple and effective model to predict the severity in patients with HFRS. A study shows that the Sequential Organ Failure Assessment (SOFA) score is related to the severity of HFRS, but this scoring system is more complex compared with other scoring systems. Besides, it does not include the clinical characteristics of patients and cannot directly reflect the severity of patients, so its clinical application is limited [8]. Nomogram is a statistical prediction model established based on the characteristic phenotype of the disease, which is used to predict the probability of a certain outcome event in a population with certain characteristics in the future. Nomogram transforms the complex regression equation into a visual graph, making the results of the prediction model more readable and convenient to evaluate the patient’s condition [9]. With this clinical prediction model, doctors can simply and accurately predict the patient’s condition, thereby providing a basis for clinical decision-making. Consequently, in this study, we retrospectively analyzed the clinical characteristics and laboratory results of HFRS patients and aimed to develop and verify a simple and applicable nomogram that predicts the severity of the patient’s condition. It will be the first nomogram of HFRS.

## 2 Methods

### 2.1 Study population

This study retrospectively analyzed a total of 155 consecutive patients diagnosed with HFRS in Jingzhou Central Hospital from January 1, 2015, to December 31, 2019. One hundred nine patients from January 1, 2015, to December 31, 2018, served as a training cohort, and 46 patients from January 1, 2019, to December 31, 2019, served as an independent verification cohort. Patients with confirmed HFRS were included in this study. The diagnostic criteria of the patients were as follows: (1) acute fever, accompanied by abnormal renal function, thrombocytopenia, etc.; and (2) the hantavirus-specific immunoglobulin (Ig) M antibody in the peripheral blood was positive. The exclusion criteria included: (1) age <8 years; (2) pregnant women; and (3) acute or chronic nephropathy and hematological diseases.

### 2.2 Data collection

Well-trained doctors extracted the patient’s demographic characteristics, basic diseases, clinical manifestations, and laboratory parameters through the electronic medical record system. Laboratory parameters included complete blood count, urine routine, procalcitonin (PCT), C-reactive protein (CRP), liver and kidney function, electrolytes, myocardial enzymes, and hantavirus-specific antibodies.

According to the clinical characteristics of patients, such as body temperature, blood pressure, urine output, edema, and renal injury indicators like urinary protein and urea nitrogen, the severity of HFRS was divided into four clinical types [10]. The four clinical types were as follows: (1) the mild group had renal injury without hypotension and oliguria; (2) the moderate group had obvious uremia, bulbar conjunctival edema, skin and mucosal hemorrhage, and acute renal failure with typical oliguria; (3) the severe group showed severe uremia, bulbar conjunctiva and peritoneal or pleural effusion, skin and mucosal bleeding, hypotension, and acute renal failure with oliguria (patients with daily output of 50–500 mL ≤5 days or urine output <100 mL/day ≤2 days); (4) the critically ill group had one or more of the following manifestations compared with the severe group: refractory shock (≥2 days), heart failure, pulmonary edema, visceral hemorrhage, cerebral edema, severe secondary infection, and severe acute renal failure with oliguria (urine volume 50–500 mL/day >5 days) or anuria (urine <100 mL/day >2 days) or blood urea nitrogen (BUN) >42.84 mmol/L. In this study, patients were divided into two groups. The mild group was composed of mild and moderate patients, while the severe group was composed of severe and critically ill patients.


**Ethics approval and consent to participate:** The study was reviewed and approved for publication by the Institutional Review Board of Jinghzou Central Hospital, and the requirement for informed consent from the study participants was waived.
**Consent for publication:** Not applicable.

### 2.3 Statistical analysis

All statistical analyses in this study were carried out using R software (version 4.0.3; http://www.r-project.org). The statistical significance levels of all reports were double tailed, and *p* < 0.05 was considered statistically significant. The R software packages involved in the implementation of R software mainly include compareGroups, glmnet, rms, pROC, rmda, and so on. The demographic characteristics, basic diseases, clinical manifestations, and laboratory parameters were statistically analyzed by compareGroups R software package, in which the Shapiro–Wilks test was performed to determine whether it was normal or nonnormal distribution. Continuous variables with a normal distribution were expressed as the mean  ±  standard deviation (SD), while nonnormally distributed continuous variables were expressed as the median (interquartile range). Categorical variables were presented as percentages (%). LASSO regression is a model in which the L1-norm constraint term is added to the cost function of the linear regression model. It is used to analyze medical data with high dimension, strong correlation, and small samples by controlling the parameter lambda for variable screening and complexity adjustment [11]. In this study, the glmnet package in LASSO regression was used to select the best predictive characteristics of risk factors from HFRS patients. Multivariate logistic regression analysis was applied to construct the nomogram of the predictive model by including the selected variables with non-zero coefficient characteristics in the LASSO regression model [12].

We evaluated the performance of the nomogram through discrimination and calibration in the training population and the verification population, respectively. Since the consistency index (C-index) is equivalent to the area under the receiver operating characteristic curve (AUC) in logistic regression, we used the AUC to evaluate the discriminative ability of the nomogram [13]. The Hosmer–Lemeshow goodness-of-fit test is performed to evaluate the calibration of the nomogram, and a calibration curve is drawn to visualize the consistency between the predicted results and the observed results [14]. By quantifying the net benefit under each risk threshold probability, the decision curve analysis (DCA) of the model is drawn to evaluate the clinical validity of the nomogram [15]. We drew a nomogram plot and a calibration plot based on the rms R package. The pROC R package was used to draw the receiver operating characteristic (ROC) curve and calculate the C-index. The rmda R package was used to draw the DCA and the clinical impact curve.

## 3 Results

### 3.1 Demographic and clinical characteristics of patients with HFRS

A total of 155 HFRS patients were included in our study, of whom 11 died, with a mortality rate of 7.10%. Table 1 summarizes the demographic characteristics of HFRS in the training cohort and the verification cohort, showing that there is no significant difference in gender, age, basic disease, clinical disease classification, and clinical outcome between the two populations. We analyzed the clinical characteristics of mild and severe groups in the training cohort of 109 patients with HFRS. The median age of the training cohort was 53 years, including 79 men and 30 women (Table 2). The most common clinical manifestations of HFRS patients were fever (90.8%), oliguria (58.7%), nausea (35.8%), chills (35.8%), vomiting (33.0%), diarrhea (28.4%), headache (26.6%), low back pain (25.7%), fatigue (22.0%), abdominal distension (20.2%), and so on. Among the aforementioned symptoms, only oliguria and arthralgia were statistically different between the critically ill group and the mild group. The results of laboratory examination showed that the levels of white blood cells (WBCs), neutrophils, lymphocytes, procalcitonin (PCT), C-reactive protein (CRP), urine protein, urea nitrogen, creatinine, cystatin C, creatine kinase, creatine kinase muscle-brain isoform (CK-MB), and myoglobin increased more significantly in severe HFRS patients, while the levels of platelets (PLT), hemoglobin (Hb), albumin, and calcium (Ca) decreased more significantly in severe patients.

**Table 1:** Baseline characteristics of patients with HFRS in the training and validation cohorts

Characteristic	All patients	Training cohort	Validation cohort	*P* value
	*N* = 155	*N* = 109	*N* = 46	
**Sex**				0.790
Female	41 (26.5%)	30 (27.5%)	11 (23.9%)	
Male	114 (73.5%)	79 (72.5%)	35 (76.1%)	
**Age, years**	54.0 (47.0–62.0)	53.0 (47.0–62.0)	55.0 (50.0–63.8)	0.323
**Basic disease**				0.139
No	109 (70.3%)	81 (74.3%)	28 (60.9%)	
Yes	46 (29.7%)	28 (25.7%)	18 (39.1%)	
**Clinical type**				0.474
Mild	69 (44.5%)	46 (42.2%)	23 (50.0%)	
Severe	86 (55.5%)	63 (57.8%)	23 (50.0%)	
**Clinical outcomes**				0.508
Deceased	11 (7.10%)	9 (8.26%)	2 (4.35%)	
Survived	144 (92.9%)	100 (91.7%)	44 (95.7%)	

Basic diseases include hypertension, diabetes, coronary heart disease, stroke, chronic liver disease, chronic lung disease, and other diseases. *P* values indicate differences between training and validation cohorts. *P* < 0.05 was considered statistically significant.

**Table 2:** Demographic and clinical features of patients with HFRS in the training cohorts

Characteristic	All patients	Mild	Severe	*P* value
	*N* = 109	*N* = 46	*N* = 63	
**Sex**				0.218
Female	30 (27.5%)	16 (34.8%)	14 (22.2%)	
Male	79 (72.5%)	30 (65.2%)	49 (77.8%)	
**Age, years**	53.0 (47.0–62.0)	50.5 (47.0–62.0)	57.0 (46.5–62.5)	0.337
**Signs and symptoms**				
***Fever***				0.186
No	10 (9.17%)	2 (4.35%)	8 (12.7%)	
Yes	99 (90.8%)	44 (95.7%)	55 (87.3%)	
***Chills***				0.428
No	70 (64.2%)	32 (69.6%)	38 (60.3%)	
Yes	39 (35.8%)	14 (30.4%)	25 (39.7%)	
***Headache***				1.000
No	80 (73.4%)	34 (73.9%)	46 (73.0%)	
Yes	29 (26.6%)	12 (26.1%)	17 (27.0%)	
***Nausea***				1.000
No	70 (64.2%)	30 (65.2%)	40 (63.5%)	
Yes	39 (35.8%)	16 (34.8%)	23 (36.5%)	
***Vomiting***				0.053
No	73 (67.0%)	36 (78.3%)	37 (58.7%)	
Yes	36 (33.0%)	10 (21.7%)	26 (41.3%)	
***Abdominal bloating***				0.917
No	87 (79.8%)	36 (78.3%)	51 (81.0%)	
Yes	22 (20.2%)	10 (21.7%)	12 (19.0%)	
***Poor appetite***				0.356
No	95 (87.2%)	38 (82.6%)	57 (90.5%)	
Yes	14 (12.8%)	8 (17.4%)	6 (9.52%)	
***Abdominal pain***				0.731
No	100 (91.7%)	43 (93.5%)	57 (90.5%)	
Yes	9 (8.26%)	3 (6.52%)	6 (9.52%)	
***Backache***				0.762
No	81 (74.3%)	33 (71.7%)	48 (76.2%)	
Yes	28 (25.7%)	13 (28.3%)	15 (23.8%)	
***Diarrhea***				0.124
No	78 (71.6%)	37 (80.4%)	41 (65.1%)	
Yes	31 (28.4%)	9 (19.6%)	22 (34.9%)	
***Dyspnea***				0.072
No	104 (95.4%)	46 (100%)	58 (92.1%)	
Yes	5 (4.59%)	0 (0.00%)	5 (7.94%)	
***Oliguria***				0.010
No	45 (41.3%)	26 (56.5%)	19 (30.2%)	
Yes	64 (58.7%)	20 (43.5%)	44 (69.8%)	
***Cough***				0.731
No	100 (91.7%)	43 (93.5%)	57 (90.5%)	
Yes	9 (8.26%)	3 (6.52%)	6 (9.52%)	
***Expectoration***				1.000
No	104 (95.4%)	44 (95.7%)	60 (95.2%)	
Yes	5 (4.59%)	2 (4.35%)	3 (4.76%)	
***Chest tightness***				0.394
No	104 (95.4%)	45 (97.8%)	59 (93.7%)	
Yes	5 (4.59%)	1 (2.17%)	4 (6.35%)	
***Black stool***				1.000
No	106 (97.2%)	45 (97.8%)	61 (96.8%)	
Yes	3 (2.75%)	1 (2.17%)	2 (3.17%)	
***Fatigue***				0.521
No	85 (78.0%)	34 (73.9%)	51 (81.0%)	
Yes	24 (22.0%)	12 (26.1%)	12 (19.0%)	
***Orbita pain***				0.261
No	106 (97.2%)	46 (100%)	60 (95.2%)	
Yes	3 (2.75%)	0 (0.00%)	3 (4.76%)	
***Myalgia***				0.163
No	100 (91.7%)	40 (87.0%)	60 (95.2%)	
Yes	9 (8.26%)	6 (13.0%)	3 (4.76%)	
***Arthralgia***				0.029
No	105 (96.3%)	42 (91.3%)	63 (100%)	
Yes	4 (3.67%)	4 (8.70%)	0 (0.00%)	
***Pulmonary hemorrhage***				0.508
No	107 (98.2%)	46 (100%)	61 (96.8%)	
Yes	2 (1.83%)	0 (0.00%)	2 (3.17%)	
***Gastrointestinal bleeding***				0.072
No	104 (95.4%)	46 (100%)	58 (92.1%)	
Yes	5 (4.59%)	0 (0.00%)	5 (7.94%)	
***Cerebral hemorrhage***				0.508
No	107 (98.2%)	46 (100%)	61 (96.8%)	
Yes	2 (1.83%)	0 (0.00%)	2 (3.17%)	
***History of rat exposure***				0.507
No	33 (30.3%)	16 (34.8%)	17 (27.0%)	
Yes	76 (69.7%)	30 (65.2%)	46 (73.0%)	
**Highest temperature, °C**	39.0 ± 0.63	39.1 ± 0.59	39.0 ± 0.65	0.192
**Time from symptom onset to admission**	5.00 (4.00–7.00)	5.00 (4.00–7.00)	5.00 (4.00–6.00)	0.173
**Laboratory findings**				
WBC, ×10^9^/L	20.5 (12.4–30.6)	12.6 (9.53–21.5)	25.2 (17.5–35.8)	<0.001
Neutrophils, ×10^9^/L	9.98 (6.41–18.7)	6.52 (4.58–10.0)	14.3 (9.30–22.2)	<0.001
Lymphocytes, ×10^9^/L	5.54 (3.69–8.13)	4.78 (3.07–6.75)	6.30 (4.06–9.25)	0.021
Hb, g/L	107 ± 20.1	115 ± 16.0	100 ± 20.5	<0.001
Platelets, ×10^9^/L	32.0 (15.0,59.0)	54.0 (35.2,93.0)	22.0 (12.0,35.0)	<0.001
Atypical lymphocyte, %	7.50 ± 5.67	6.67 ± 4.39	8.10 ± 6.42	0.173
PCT, ng/mL	3.12 (1.00–7.46)	1.21 (0.60–2.15)	6.16 (2.26–10.7)	<0.001
CRP, mg/L	42.9 (23.5–56.0)	30.9 (19.2–49.9)	51.0 (32.3–73.6)	0.001
**Urine protein**				0.002
1+	12 (11.0%)	8 (17.4%)	4 (6.35%)	
2+	34 (31.2%)	18 (39.1%)	16 (25.4%)	
3+	44 (40.4%)	19 (41.3%)	25 (39.7%)	
4+	19 (17.4%)	1 (2.17%)	18 (28.6%)	
Albumin, g/L	26.8 (23.8–29.8)	27.9 (24.8–31.5)	25.7 (23.2–28.9)	0.022
ALT, U/L	62.9 (41.7–108)	59.2 (43.0–110)	72.2 (41.7–106)	0.556
AST, U/L	104 (67.7–184)	82.7 (58.2–158)	115 (77.9–222)	0.051
TBIL, μmol/L	13.9 (10.8–19.0)	12.9 (10.8–17.0)	14.7 (11.2–23.9)	0.114
DBIL, μmol/L	5.30 (3.80–8.00)	4.70 (3.70–5.77)	6.20 (4.20–10.5)	0.006
Urea nitrogen, mmol/L	21.8 (14.4–28.5)	14.2 (10.1–18.7)	26.7 (21.9–31.6)	<0.001
Creatinine, μmol/L	483 (220–616)	215 (142–309)	604 (514–723)	<0.001
Uric acid, μmol/L	596 (485–713)	616 (498–699)	594 (484–744)	0.927
Cystatin C, mg/L	3.73 (2.32–4.53)	2.32 (1.81–3.11)	4.37 (3.72–6.12)	<0.001
Ca, mmol/L	1.72 ± 0.21	1.85 ± 0.16	1.62 ± 0.19	<0.001
K, mmol/L	4.65 ± 0.70	4.27 ± 0.57	4.94 ± 0.65	<0.001
P, mmol/L	0.75 (0.46–0.98)	0.89 (0.74–1.06)	0.53 (0.38–0.83)	<0.001
Creatine Kinase, U/L	184 (87.8–376)	111 (69.4–206)	211 (124–408)	0.002
CK-MB, U/L	40.4 (24.7–54.6)	33.1 (18.3–50.0)	45.1 (27.9–71.2)	0.001
cTnI, μg/L	0.05 (0.01–0.31)	0.02 (0.01–0.80)	0.06 (0.02–0.21)	0.085
Myoglobin, μg/L	166 (58.9–289)	65.3 (47.7–281)	236 (92.2–377)	<0.001

*P* values indicate differences between mild and severe groups. *P* < 0.05 was considered statistically significant. Abbreviations: WBC, white blood cell; Hb, hemoglobin; PCT, procalcitonin; CRP, C-reactive protein; ALT, alanine aminotransferase; AST, aspartate aminotransferase; TBIL, total bilirubin; DBIL, direct bilirubin; Ca, calcium; K, potassium; P, phosphorus; CK-MB, creatine kinase muscle-brain isoform; cTnI, cardiac troponin I.

### 3.2 Prognostic factors in patients with severe HFRS

**Figure 1:**
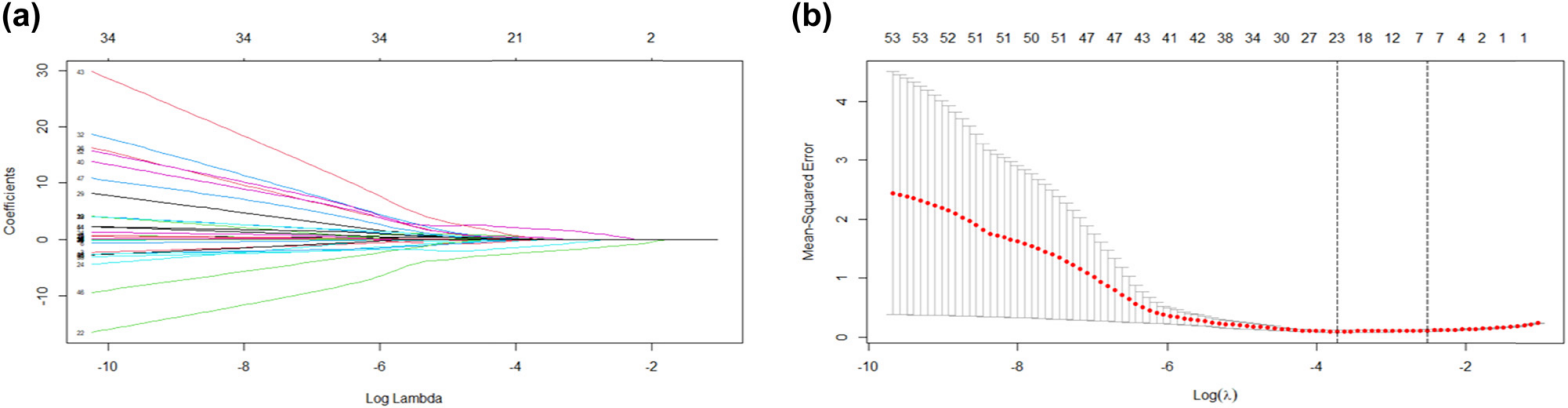
Predictive factors for patients with severe HFRS were selected by LASSO regression. (a) Fifty-four variables from the training cohort were included in the LASSO regression (*y*-axis). The average number of predictors was shown at the top *x*-axis. (b) The parameter lambda (*λ*) was selected by using tenfold cross-validation based on the minimum standard in the LASSO model. The two vertical dashed lines represent the log(*λ*) of the minimum mean square error (left dashed line) and the log(*λ*) of the minimum distance standard error (right dashed line). HFRS, hemorrhagic fever with renal syndrome; LASSO, least absolute shrinkage and selection operator; *λ*, lambda.

**Figure 2:**
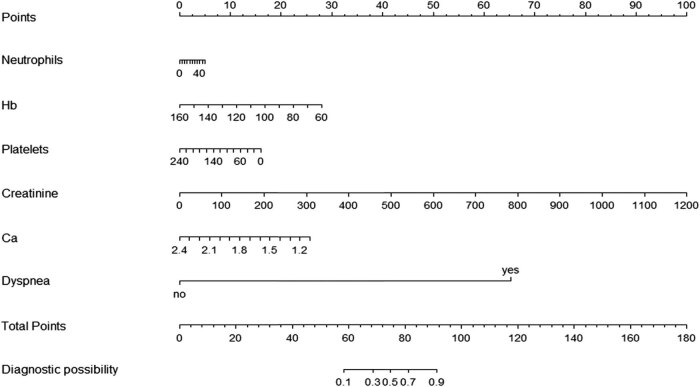
Nomogram to predict the risk of severity in patients with HFRS. To use the nomogram in clinical practice, a line can be drawn up to calculate the patient’s total score by the value of each predictor variable, and then, a line can be drawn down based on the total score to find out the possibility of severe HFRS. HFRS, hemorrhagic fever with renal syndrome; Hb, hemoglobin; Ca, calcium.

After excluding variables with irrelevant characteristics from the training cohort, 54 variables were finally included in the LASSO regression for analysis (Figure 1a). The parameter lambda (*λ*) was selected by using tenfold cross-validation based on the minimum standard in the LASSO model. The two vertical dashed lines in Figure 1b represent the log(*λ*) of the minimum mean square error (left dashed line) and the log(*λ*) of the minimum distance standard error (right dashed line). To provide a simple and accurate clinical model, six variables corresponding to the log(*λ*) of minimum mean square error, “neutrophils,” “Hb,” “Platelets,” “Creatinine,” “Ca,” and “Dyspnea,” were selected into the model (Figure 2, Table 3).

**Table 3:** Prognostic factors in patients with severe HFRS

Intercept and variable	*β*	Odds ratio (95% CI)	*P* value
Intercept	4.437	84.523 (0.001–3.508 × 10^7^)	0.465
Neutrophils	0.013	1.013 (0.913–1.139)	0.811
Hb	−0.037	0.963 (0.916–1.004)	0.103
Platelets	−0.009	0.991 (0.965–1.0140)	0.481
Creatinine	0.011	1.011 (1.007–1.017)	0.001
Ca	−2.632	0.072 (0.000–16.208)	0.361
Dyspnea	18.937	1.676 × 10^8^ (0.000–NA)	0.994

Abbreviations: HFRS, hemorrhagic fever with renal syndrome; *β*, regression coefficient; CI, confidence interval; Hb, hemoglobin; Ca, calcium; NA, not applicable.

### 3.3 Development and verification of a nomogram

**Figure 3:**
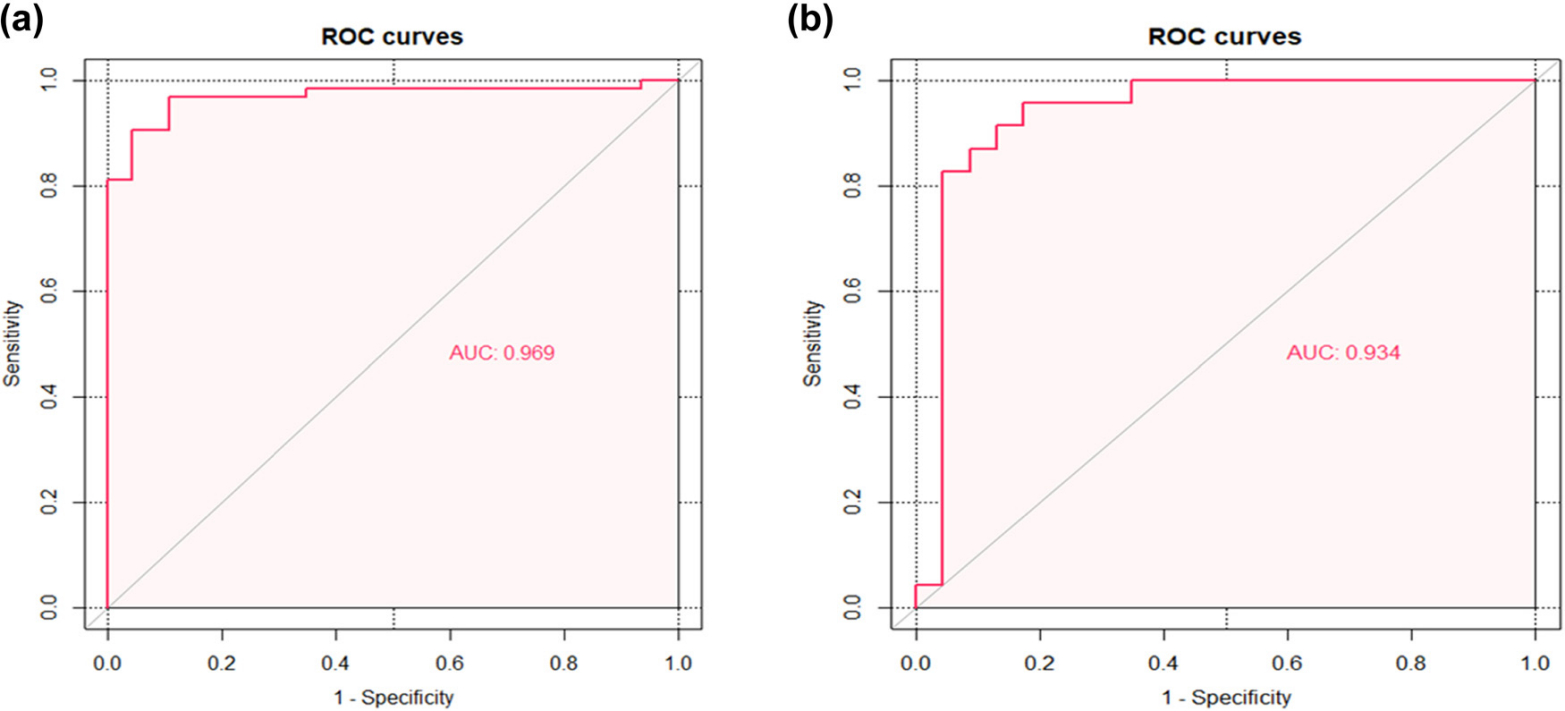
ROC curve to evaluate the discriminative performance of the nomogram in the training and validation cohorts. (a) Training cohort. (b) Validation cohort. ROC, receiver operating characteristic.

**Figure 4:**
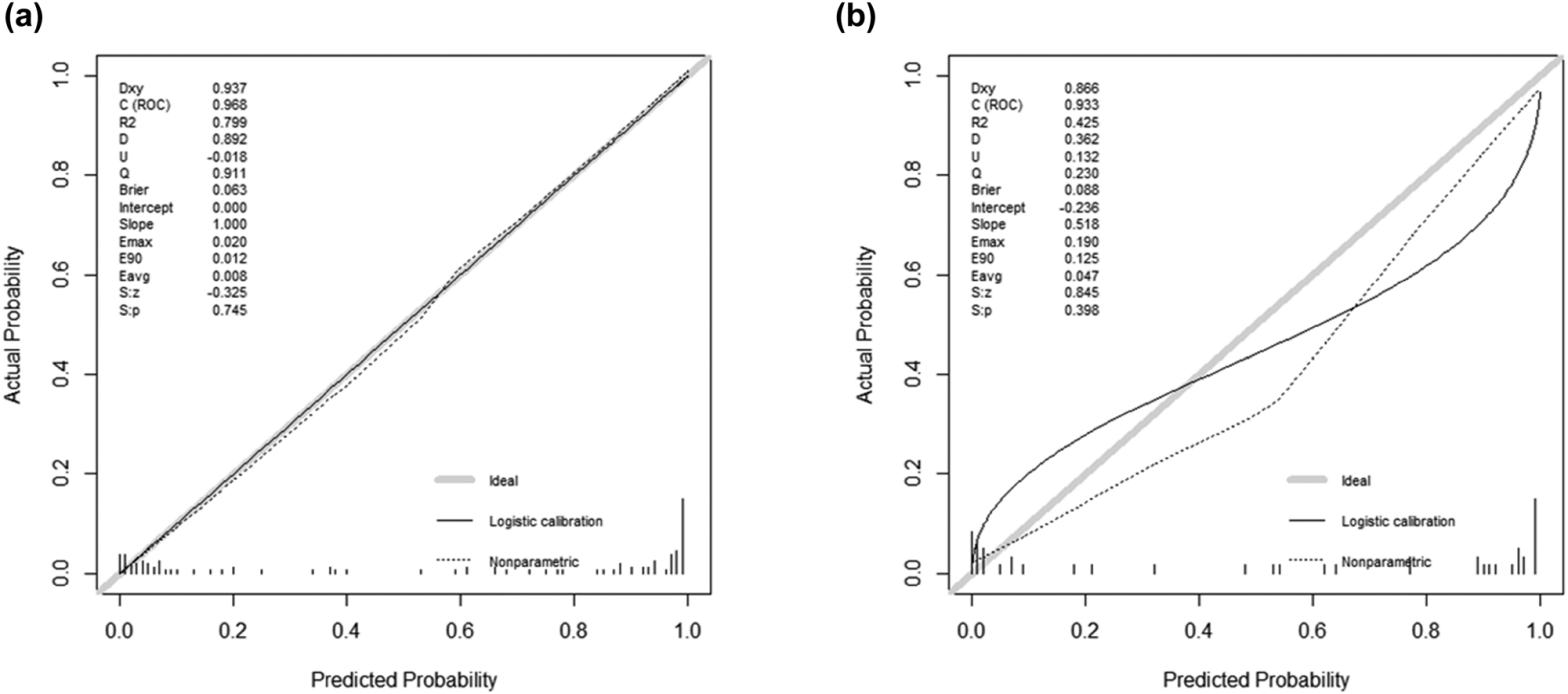
Calibration curves for training and validation of the nomogram. (a) Training cohort. (b) Validation cohort. The *x*-axis represents the nomogram-predicted probability and the *y*-axis represents the actual probability of severe HFRS. The black solid line represents the predictive performance of the nomogram, and the diagonal gray line represents the ideal nomogram model. HFRS, hemorrhagic fever with renal syndrome.

The regression model based on six independent variables for predicting the severity of HFRS determined by LASSO regression analysis was represented by a nomogram (Figure 2). According to the nomogram, we can get the points corresponding to each predictor and then record the total score of these points, so as to accurately predict the risk of serious illness in the corresponding HFRS patients. As shown in Figure 3a and b, the AUC of the nomogram in the training and validation cohorts is 0.969 (95% CI: 0.935–1.000) and 0.934(95% CI: 0.847–1.000), respectively. The AUC values of these two cohorts are more than 0.9, indicating that the model has good discrimination. In the training cohort and the validation cohort, the calibration plot and Hosmer–Lemeshow goodness-of-fit test showed that the *P* values were 0.745 and 0.398, respectively; both *P* values were >0.05, demonstrating that the predicted probability of nomogram was in good agreement with the real results (Figure 4a and b).

### 3.4 Clinical utility

**Figure 5:**
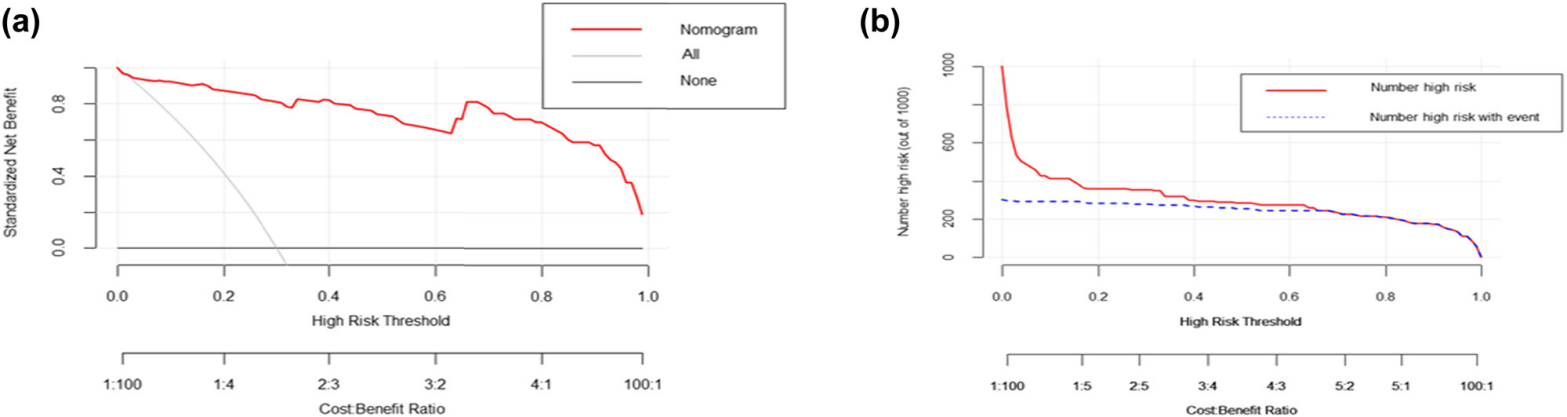
The decision curve and clinical impact curve analysis of the nomogram for predicting severe HFRS. (a) The DCA compares the clinical net benefits of scenarios that predict the probability of severe HFRS: a perfect predictive model (solid grey line), no screening (horizontal solid black line), and screening based on the nomogram (solid red line). The *y*-axis measures the net benefit. DCA shows that using nomogram to predict the risk of severe HFRS can benefit patients if the threshold probability of the patient or doctor is between 0 and 1. (b) Clinical impact curve of the nomogram plots the number of HFRS patients classified as high risk, and the number of cases classified as high risk with the event at each risk threshold. HFRS, hemorrhagic fever with renal syndrome; DCA, decision curve analysis.

DCA shows that using nomogram to predict the risk of severe illness in HFRS patients can benefit patients if the threshold probability of the patient or doctor is between 0 and 1 (Figure 5a). Within this range, according to the nomogram, the net benefit is comparable, but there are multiple overlaps.

## 4 Discussion

HFRS is an infectious disease of global concern caused by hantavirus infection, which is characterized by increased vascular permeability, acute thrombocytopenia, and renal damage. China has recorded the highest number of confirmed HFRS cases in the world [3]. HFRS patients can be clinically manifested as mild, moderate, severe, and critical. Generally, HFRS caused by *HTNV* and *SEOV* infection is more serious, with a mortality rate of 5–15% [7]. The purpose of this study is to analyze the clinical characteristics and laboratory examination of patients with HFRS and establish a nomogram to predict the severity of the disease. Through this simple and feasible prediction model, we can identify the patient’s condition early and provide patients with better medical measures promptly to reduce patient mortality.

The typical course of HFRS can be divided into five different stages: fever, hypotension, oliguria, polyuria, and recovery. In the hypotension stage, one-third of the deaths of HFRS patients are related to irreversible shock, and thrombocytopenia and leukocytosis are the characteristics of this stage. Thrombocytopenia can cause petechiae of the skin or mucous membranes, conjunctival congestion, hematemesis, hemoptysis, hematuria, and fatal intracranial hemorrhage [16]. In addition, platelet dysfunction may also lead to abnormal blood coagulation [17]. In the training cohort (Table 2), there were 63 seriously ill patients, including 2 patients with pulmonary hemorrhage, 5 patients with gastrointestinal hemorrhage, and 2 patients with intracranial hemorrhage. However, there is no statistical difference between severe and mild patients due to the small sample size.

In this study, the platelet count decreased more significantly in the severe group. At the same time, after the parameter *λ* was selected by the tenfold cross-validation based on the minimum standard in the LASSO model, the platelet count was also included in the regression model, indicating that platelet count can be used as a predictor of the severity of HFRS patients.

In patients with viral hemorrhagic fever, platelets can cause abnormal homeostasis and inflammatory activation, thereby inhibiting the body’s antiviral immune response and thus making patients have a high level of viremia. This mechanism leads to the aggravation of the patient’s condition [18]. Other studies have shown that WBC, PLT, platelet distribution width (PDW), and PCT can be used as valuable parameters for the severity of HFRS patients, especially the change of PDW on the first day of hospitalization is related to the survival rate of severe HFRS patients and can be used as a potential predictor [19]. In this study, the increase of WBC in patients with severe HFRS was significantly higher than that in mild patients, whereas a study showed that compared with leukocytosis, thrombocytopenia may better predict the prognosis of severe acute kidney injury (AKI) in patients with acute *HTNV* infection [20]. Neutrophil activation is usually common in bacterial infections. It is interesting to note that markers of neutrophil activation, such as myeloperoxidase (MPO), human neutrophil elastase (HNE), histone, and interleukin-8 (IL-8), are significantly increased in the blood and tissue of patients with severe HFRS. These results suggest that neutrophils can be activated by endothelial cells infected by hantavirus and may help to determine the degree of renal pathological damage in patients with severe HFRS [21]. In our study, neutrophil in patients with severe HFRS was also higher than that in mild patients, which may further support this view from a clinical perspective.

Acute renal failure can occur in patients with severe HFRS, usually caused by tubulointerstitial and glomerular damage [22]. In addition, the increase of platelet production and platelet activation may cause intravascular coagulation, the accumulation of inflammatory cells, and the release of proinflammatory cytokines in the kidney tissue, which can also lead to kidney damage [23,24]. In this study, renal function impairment indicators such as urine protein, urea nitrogen, creatinine, and cystatin C were significantly increased in severe HFRS patients. Previous studies have also confirmed that plasma cystatin C and alpha-1-microglobulin (A1M) can be used as early and sensitive markers of renal injury in patients with HFRS and can predict AKI [25,26]. The complexity adjustment of LASSO regression model is controlled by the parameter *λ* to avoid overfitting. The larger the *λ*, the greater the penalty for a linear model with more variables, and a model with fewer variables is finally obtained [11]. So, in the end, only creatinine is included in the prediction model. Patients present with acute renal failure are often accompanied by hypocalcemia. Wang et al. [27] studied the prognostic ability of serum calcium in patients with severe AKI, and the results showed that low Ca concentration was an independent predictor of all-cause mortality in patients with severe AKI. Similarly, in our study, the average serum calcium concentration in HFRS patients was lower than the normal level, especially in severely ill patients.

In addition, patients with HFRS can also experience acute cardiovascular events such as acute myocardial infarction and stroke, indicating that the increased levels of myocardial injury indicators such as creatine kinase, CK-MB, and myoglobin can predict the risk of disease progression in patients [28]. Another study showed that hypoproteinemia in patients with acute HFRS was associated with the severity of the patient’s disease, which is consistent with our findings [29]. The clinical manifestations of HFRS patients are diverse, including fever, headache, fatigue, myalgia, back pain, and so on [30]. In addition to the aforementioned symptoms in this study, gastrointestinal symptoms such as nausea, vomiting, diarrhea, abdominal distension, and respiratory symptoms such as cough and dyspnea were also manifested. Severe HFRS patients may initially present with dry cough, followed by tachycardia, dyspnea, and then may rapidly progress to noncardiogenic pulmonary edema, hypotension, and circulatory failure, with a case-fatality rate of about 45% [31].

On the basis of LOSSA regression, we finally included six predictive indicators: “neutrophils,” “Hb,” “platelets,” “creatinine,” “Ca,” and “dyspnea” to establish a nomogram. The AUC value of the nomogram is greater than 0.9 in both the training cohort and the verification cohort, indicating that the predictive model has a high value. Both the calibration plot and the Hosmer–Lemeshow goodness-of-fit test show that the prediction probability of the nomogram is in good agreement with the real results. In addition, to evaluate the clinical effectiveness of nomogram, we applied DCA to provide observations of clinical results based on threshold probability, from which net benefits can be derived (net benefit is defined as the proportion of true positives minus the proportion of false positives, weighted by the relative harm of false-positive and false-negative results) [15,32]. In this study, if the threshold probability of the patient or doctor is between 0 and 1, the use of the nomogram to assess the risk of severe illness in HFRS patients can benefit patients. The clinical impact curve also intuitively shows that the nomogram has a better overall net benefit within a wide range of threshold probability and affecting the prognosis of patients.

However, our research also has some limitations. First, it is designed to be retrospective, and the inherent limitations of this type of research inevitably affect the choice of patients. Second, although we collected patient data from different periods to validate the model, it came from a single center. If possible, we still need cohorts from other research centers to validate the model. Finally, the number of cases in our study is relatively small, which may weaken the predictive ability of the current model.

## 5 Conclusion

This study developed and verified a novel nomogram for predicting the condition of patients with HFRS, which is the first nomogram used to predict HFRS. On the basis of these six laboratory and clinical parameters, clinicians can easily and accurately assess the individual risk of HFRS patients, make correct clinical decisions, and provide the best treatment for patients.

## Abbreviations

HFRS: hemorrhagic fever with renal syndrome
LASSO: least absolute shrinkage and selection operator
Hb: hemoglobin
Ca: calcium
AUC: area under the receiver operating characteristic curve
CI: confidence interval
*HTNV*: *Hantaan virus*
*DOBV*: *Dobrava virus*
*SEOV*: *Seoul virus*
*AMV*: *Amur virus*
*PUUV*: *Puumala virus*
SOFA: sequential organ failure assessment
Ig: immunoglobulin
PCT: procalcitonin
CRP: C-reactive protein
BUN: blood urea nitrogen
SD: standard deviation
C-index: consistency index
DCA: decision curve analysis
ROC: receiver operating characteristic
WBC: white blood cell
CK-MB: creatine kinase muscle-brain isoform
PLT: platelet
*λ*: lambda
PDW: platelet distribution width
AKI: acute kidney injury
MPO: myeloperoxidase
HNE: human neutrophil elastase
IL-8: interleukin-8
A1M: alpha-1-microglobulin
